# Effective doses for common paediatric diagnostic general radiography examinations at a major Australian paediatric hospital and the communication of associated radiation risks

**DOI:** 10.1002/jmrs.632

**Published:** 2022-12-01

**Authors:** Victoria J. Earl, Amanda O. G. Potter, Amanda A. Perdomo

**Affiliations:** ^1^ Department of Medical Imaging The Royal Children's Hospital Parkville Victoria Australia; ^2^ Department of Medical Imaging The Royal Melbourne Hospital Parkville Victoria Australia

**Keywords:** Effective dose, general radiography, paediatric, radiation risk, risk communication

## Abstract

**Introduction:**

Health professionals in paediatric medical imaging are routinely required to communicate radiation risks to carers and patients. Effective dose alone cannot be used to specify and communicate the radiation risk for an individual as risks are dependent on many factors including age and patient sex. In this study, we estimated typical effective doses for 20 commonly performed paediatric general radiography examinations using the weight‐based imaging protocols employed at a major Australian specialist paediatric hospital. Effective doses were used to estimate and categorise associated age‐based stochastic risks with commonly used risk terminology to facilitate communication of risk.

**Methods:**

Paediatric protocols for common general radiography examinations and World Health Organization 50th percentile weight‐for‐age data for females and males aged up to 18 years were used to estimate typical effective doses using Monte Carlo software and lifetime risk of cancer incidence using published data. Results were used to determine standardised levels of risk using the Calman risk model.

**Results:**

Effective doses, corresponding lifetime risk of cancer incidence and level of risk category from 20 general radiography examinations for paediatric patients were calculated and presented for ease of communication. Doses ranged from <0.001 mSv (negligible risk) to 1.6 mSv (low risk).

**Conclusion:**

Typical effective doses from common paediatric general radiography examinations, the associated lifetime risk of cancer incidence and level of risk have been established for our institution. This can be used to convey risks to health professionals, patients and carers in ways that are easy to understand and compare with other everyday risks.

## Graphical abstract

The typical effective doses for 20 of the most commonly performed paediatric general radiography examinations were estimated using the weight‐based imaging protocols employed at our institution. The effective doses were used to estimate and categorise associated age‐based stochastic risks with commonly used risk terminology to facilitate the communication of risk to patients and carers in ways that are easy to understand and compare with other everyday risks.

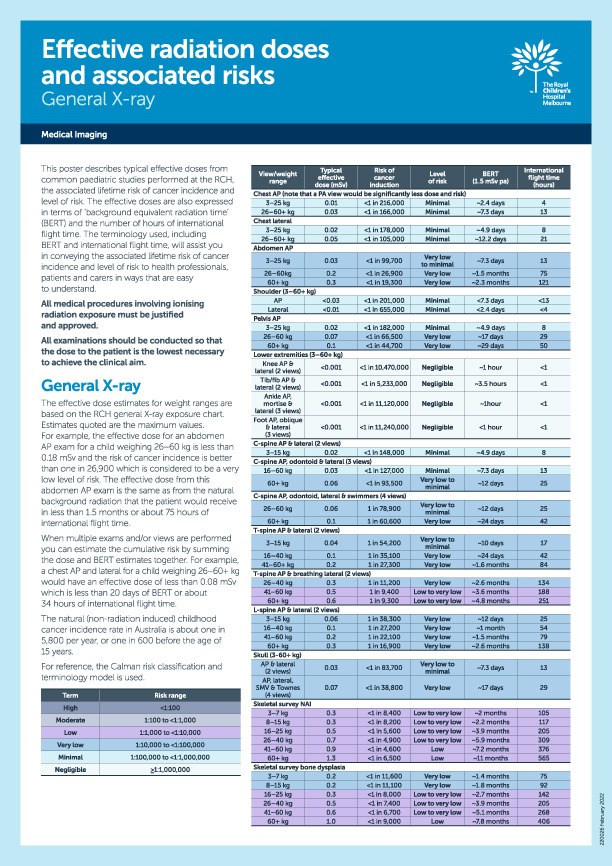

## Introduction

Diagnostic general radiography examinations are performed routinely in children, providing important information for the diagnosis, staging, treatment and follow‐up of a range of conditions and diseases. The Royal Children's Hospital, Melbourne, Australia (RCH) has five fixed general radiography rooms using indirect digital detector technology (Shimadzu RADSpeed Pro UD 150B‐10 with Canon CXDI‐70C CsI(CsI:Tl) wireless digital detector) installed in 2011. The Medical Imaging department provides a 24‐hour service. In the period January 2021 to December 2021, the Medical Imaging department imaged over 28,000 patients and acquired over 33,000 radiographs. The most commonly performed general radiography service was extremity imaging (44%) followed by chest imaging (38%). Whilst the effective doses from general radiography examinations are typically lower than those received from computed tomography (CT), fluoroscopic and nuclear medicine procedures, the radiation exposure must still be justified.^1^ In addition to evaluating the clinical relevance and appropriateness of a requested radiological examination, the justification process also requires the radiation medical practitioner (radiologist) or the operator (radiographer) to understand the magnitude of the effective doses and the associated risks (e.g. cancer induction) for the patient. It is widely accepted that the paediatric population is more radiosensitive than the adult population, and therefore paediatric age‐specific information is required for the justification process as well as communicating the magnitude of the radiation dose and the associated risk to patients and their carers.^1^, ^2^, ^3^, ^4^

Whilst there is published data regarding effective doses for paediatric general radiography examinations,^5^, ^6^, ^7^, ^8^, ^9^ as the major specialist paediatric hospital and designated state‐wide major trauma centre for paediatrics in Victoria, Australia, the Medical Imaging department is frequently approached by government departments and other hospitals for our typical effective doses for commonly performed examinations. There are currently no Australian national Diagnostic Reference Levels (DRLs) published for paediatric, or adult, general radiography examinations.

At our institution, a weight‐based exposure chart is employed to ensure a certain level of image quality is achieved given the large variation in patient weight and height for any given age. Stochastic risk is based on a patient's age so an age–weight relationship needs to be used to be able to estimate and communicate risk meaningfully.

As previously discussed by the authors,^10^ effective dose alone cannot be used to specify and communicate the associated stochastic risk for an individual examination due to differences in risk due to age and patient sex. In an earlier publication, the authors established typical effective doses from common paediatric nuclear medicine and positron emission tomography (PET) studies performed at the RCH and the associated lifetime risk of cancer incidence and level of risk for female and male children from 0 to 18 years old.^10^ Various methods are in use to categorise relative risks, with the classifications and terminology proposed by Calman being frequently used by State and National authorities such as the Australian Radiation Protection and Nuclear Safety Agency information sheets and Codes.^11^, ^12^, ^13^, ^14^ A common method is to equate the procedural effective dose to ‘background equivalent radiation time’, referred to as BERT.^15^ In Australia, natural BERT is approximately 1.5 mSv per annum.^12^ It is also potentially misleading as BERT is a whole‐body exposure over a time period, whereas a diagnostic procedure involves a radiation exposure delivered in a very short amount of time and often concentrated to parts of the body, and there may be a dose and dose rate effectiveness factor involved which is currently unknown. Another approach compares the radiation risk with odds of dying from more everyday hazards such as driving a car, riding a bike or air travel.^16^, ^17^, ^18^, ^19^

The majority of patients, carers and some health professionals are not radiation‐literate. Using too many technical terms and complicating the narrative may increase confusion and concern in the audience.^20^ The ability to accurately and meaningfully communicate radiation risk information to health professionals, patients and carers is of utmost importance as it can influence healthcare pathway decisions. This may include referrers being hesitant to request imaging required and parents and carers hesitant to consent which can prolong the imaging process or introduce a level of concern that is not warranted. As concluded by Weider, *for effective radiation risk communication we need to personalise communication like we personalise medical diagnosis and treatment*.^20^

The primary aim of this study is to estimate the radiation dose and associated risk for the most commonly performed paediatric radiographic examinations at our institution. The secondary aim is to provide clear and accurate information to assist medical imaging professionals and other clinical staff, in the communication of radiation dose and associated risk in the paediatric setting using common risk classifications and terminologies. The standardised information can then be personalised by health professionals as required (e.g. when a patient requires multiple chest radiographs with multiple views and the overall risk needs to be estimated).

## Materials and Methods

Age‐dependent effective doses for the 20 of the most commonly performed general radiography examinations with one or more views at RCH (Table 1) for children aged 0–18 years were estimated using the RCH exposure chart (Table 2) and the PC Program for X‐ray Monte Carlo (PCXMC) software (version 2.0.1.4, STUK (Radiation and Nuclear Safety Authority, Finland)) with International Commission on Radiological Protection (ICRP) 103 tissue weighting factors.^1^ Mobile examinations were not included in this work; however, the exposure chart is the same for mobile general radiography at this institution.

**Table 1:** Commonly performed paediatric general radiography procedures at RCH. Note that not all views listed for a procedure may be performed if they are not clinically relevant for the patient. All views were included to estimate the maximum effective dose and associated risk to the patient.

Exam	Comment
Chest AP^†^	
Chest Lateral	
Abdomen AP	
Shoulder AP	
Shoulder Lateral	
Pelvis AP	
Knee AP and Lateral	
Tibia/Fibula AP and Lateral	
Ankle AP, Mortise and Lateral	
Foot AP, Oblique and Lateral	
Cervical Spine AP and Lateral	3–15 kg weight range only
Cervical Spine AP, Odontoid and Lateral	16–60+ kg weight range only
Cervical Spine AP, Odontoid, Lateral and Swimmers	26–60+ kg weight range only
Thoracic Spine AP and Lateral	
Thoracic Spine AP and Breathing Lateral	26–60+ kg weight range only
Lumbar Spine AP and Lateral	
Skull AP and Lateral	
Skull AP, Lateral, Townes and SMV^‡^	
Skeletal Survey – Non‐Accidental Injury	Skull AP, Skull Lateral, AP Chest (for ribs), Oblique Ribs Bilateral, AP Abdomen incl. Pelvis, Lateral Spine, Lateral Sternum, Bilateral AP Femur, Bilateral AP Tibia/Fibula, Bilateral Feet, Bilateral AP Humeri, Bilateral AP Forearms, Bilateral AP Hands
Skeletal Survey – Bone Dysplasia	Skull AP, Skull Lateral, AP Chest (for ribs), AP Abdomen incl. Pelvis, Lateral Spine, Bilateral AP Femur, Bilateral AP Tibia/Fibula, Bilateral Feet, AP Humerus, Bilateral AP Forearms, AP Hand

^†^ Anterior–Posterior.

^‡^ Submentovertex.

**Table 2:** Exposure chart for commonly performed paediatric general radiography procedures at RCH. Shaded cell indicates exam performed with grid. Source to detector distance 100 cm out of bucky, 110 cm with grid and or in bucky (chest radiographs at 125 cm).

Exam	kVp Range	mAs					
		3–7 kg	8–15 kg	16–25 kg	26–40 kg	41–60 kg	60+ kg
Chest AP^†^/PA^‡^	70–90	0.71	0.8	1	1.2	1.4	2
Chest Lateral	70–100	1	1.6	2	2.5	3.2	4
Sternum Lateral	65–75	4	6	12	16	20	32
Chest AP for Ribs	65–70	4	4.5	8	12	14	20
Ribs Bilateral Oblique	65–70	4	4.5	8	12	14	20
Abdomen AP	60–70	0.8	1	2	8	16	25
Abdomen AP (including pelvis)	70–75	4	5	8	10	14	25
Shoulder AP	55–65	1	1.4	2	4	10	14
Shoulder Lateral	60–70	1.6	2	3	4	14	18
Humerus AP	55–65	1.2	1.4	1.6	2	2.5	3
Forearm AP	52–55	1	1.4	1.6	2	2.5	3
Hands AP	50–55	1	1.2	1.2	1.4	1.6	2
Pelvis AP	70–75	1.4	1.6	2	8	10	20
Femur Bilateral AP	55–65	1	1.4	1.6	2	8	10
Knee AP/Lateral	55–65	1	1.2	1.4	2	2.4	3.2
Tibia/Fibula AP/Lateral (including bilateral)	55–65	1	1.2	1.4	2	2.4	3.2
Ankle AP/Mortise/Lateral	55	1	1.2	1.2	1.6	2	3
Foot AP/Oblique/Lateral	50–55	0.8	1	1	1.4	1.6	2
Cervical Spine AP/Odontoid	65–75	2	2.5	3	4	5	10
Cervical Spine Lateral	65–75	2	2.5	3	4	5	10
Cervical Spine Swimmers	77–85	‐	‐	‐	20	30	40
Thoracic Spine AP	65–70	4	4.5	8	12	14	20
Thoracic Spine Lateral	65–75	4	6	12	16	20	32
Thoracic Spine Breathing Lateral	70–75	‐	‐	‐	120	140	160
Lateral Spine AP	70–75	4	5	8	10	14	25
Lateral Spine Lateral	72–85	4	6	12	14	20	40
Skull AP/Lateral/Townes	65–75	6	8	10	12	14	16
Skull SMV^§^	65–75	8	10	12	14	16	18

^†^ Anterior–Posterior.

^‡^ Posterior–Anterior.

^§^ Submentovertex.

The views listed in Table 1 were included to conservatively estimate the maximum effective dose and associated risk to the patient, however, in practice only the views that are clinically required would be performed. Examinations of the upper extremities could not be estimated using PCXMC due to the limitations of the program's phantoms. Consequently, dosimetry was estimated for upper extremities using published data that was adjusted for the exposure parameters used at the RCH by normalising for differences in mAs and kVp, that is calculating the ratio of the parameter (linear relationship for mAs, to the power of 2 for kVp) and multiplying the derived factor to the published dose.^21^

The 50th percentile weight‐for‐age and body mass index (BMI) values published by the World Health Organization (WHO) in 2007 for female and male children from 0 to 18 years old were used to represent the average weight for each age.^22^ Weight‐for‐age reference data were not available beyond age 10 so the weight was estimated based on the WHO published data for BMI and height for ages 11–18 (weight = BMI/square of the height).^22^

Calculations and statistical analyses were performed using Microsoft Excel (Microsoft 365). The effective doses were calculated for the weight ranges 3–7, 8–15, 16–25, 26–40, 41–60 kg and 60+ kg using the corresponding anthropomorphic PCXMC phantom (0, 1, 5, 10 and 15 years modified to 176.1 m and 67.3 kg to reflect the median height and weight data for an 18 year old male, respectively). The PCXMC phantom for a 15‐year‐old (168.1 m and 56.3 kg) was suitable for use to model an 18‐year‐old female using the median WHO data (163.1 m and 56.7 kg). The adult phantom (178.6 m and 73.2 kg) was too large to model the median 18‐year‐old male and such as the decision was made to modify the 15‐year‐old phantom for the dosimetry. The RCH general radiography exposure chart provides the recommended kVp and mAs based on these weight ranges (Table 2). As noted in ICRP Publication 135, weight bands are recommended for establishing DRLs given that individual patient size does not correlate well with patient age.^23^ The median weight for male and female children was used to estimate the typical kVp and mAs used for each patient sex as per Table 2. For example, a chest anterior–posterior radiograph of a newborn female weighing 3.2 kg would have the lower exposure parameters of 70 kVp and 0.71 mAs, a 20 kg male or female 6 year old would use 75 kVp and 1 mAs and a 65 kg male 17 year old would use the upper exposure parameters of 90 kVp and 2 mAs.

Using the same approach as the authors' previous publication,^10^ the lifetime attributable risk of cancer incidence was estimated using the calculated effective doses and data provided in the Biological Effects of Ionising Radiation VII (BEIR VII) report, linearly interpolating for risk estimates for the in‐between ages.^2^ BEIR VII provides lifetime risk estimates for cancer incidence and cancer mortality resulting from a single dose of 0.1 Gy (100 mSv) at several specific ages for males and for females (Tables 12D‐1 and 12D‐2 in the BEIR VII report). These lifetime risk estimates can be used for other exposure scenarios such as the effective doses from the general radiography examinations calculated in this work. The risk of cancer incidence (all cancers) is higher than the risk of cancer mortality (all cancers) and as such cancer incidence is used here for the classification and communication of risk. For example, using data from BEIR VII, if 100,000 5‐year‐old male persons were exposed to a single dose of 0.1 Gy, it is expected that there would be an additional 1816 cases of cancer incidence (all cancers). Therefore, the risk from exposure to 1 mSv as a 5‐year‐old male is estimated as (0.001 Sv/0.1 Sv) × 1816 = 18.2 excess cancer incidence per 100,000 or 0.0182%. This can also be expressed as a ‘1 in …’ statement which in this example is 1 in 1/(18.2/100,000) = 1 in 5507.

Calman's risk classification and terminology was used to categorise the level of risk (i.e. risk of cancer incidence, e.g. 1 in 5507) by comparing the level of risk with the published classifications (Table 3).^13^

**Table 3:** Risk classifications and terminology proposed by Calman.^13^

Terminology	Risk Range
Negligible	Less than 1 in 1000,000
Minimal	1 in 100,000–1 in 1000,000
Very Low	1 in 10,000–1 in 100,000
Low	1 in 1000–1 in 10,000
Moderate	1 in 100–1 in 1000
High	More than 1 in 100

The BERT was calculated using the typical effective dose for each examination and the Australian natural background radiation dose of 1.5 mSv per annum.^12^ The BERT was converted into hours, days or months as appropriate for communication with patients and carers.

$$\text{BERT}=\text{typical effective dose}\;\left(\mathrm{mSv}\right)/1.5\;\mathrm{mSv}\;\mathrm{per}\;\text{annum}.$$

The equivalent in international flight time was calculated using the typical effective dose for each examination, the quoted effective dose for a return flight between Melbourne and London of 0.11 mSv, and a typical return flight time of 46 hours.^24^ The international flight time was converted into hours for communication with patients and carers.

$$\begin{array}{c} \text{International Flight Time}\;\left(\text{hours}\right)\\ \;=\text{typical effective dose}\;\left(\mathrm{mSv}\right)\\ \;/0.00239\;\left(\mathrm{mSv}/\text{hour}\right). \end{array}$$

The statistical analysis consisted of determining the maximum effective dose and associated BEIR VII risk and Calman risk category of the female and male estimates for the previously stated weight ranges. The maximum value in each weight range was used to conservatively represent that weight range.

## Results

**Figure 1:**
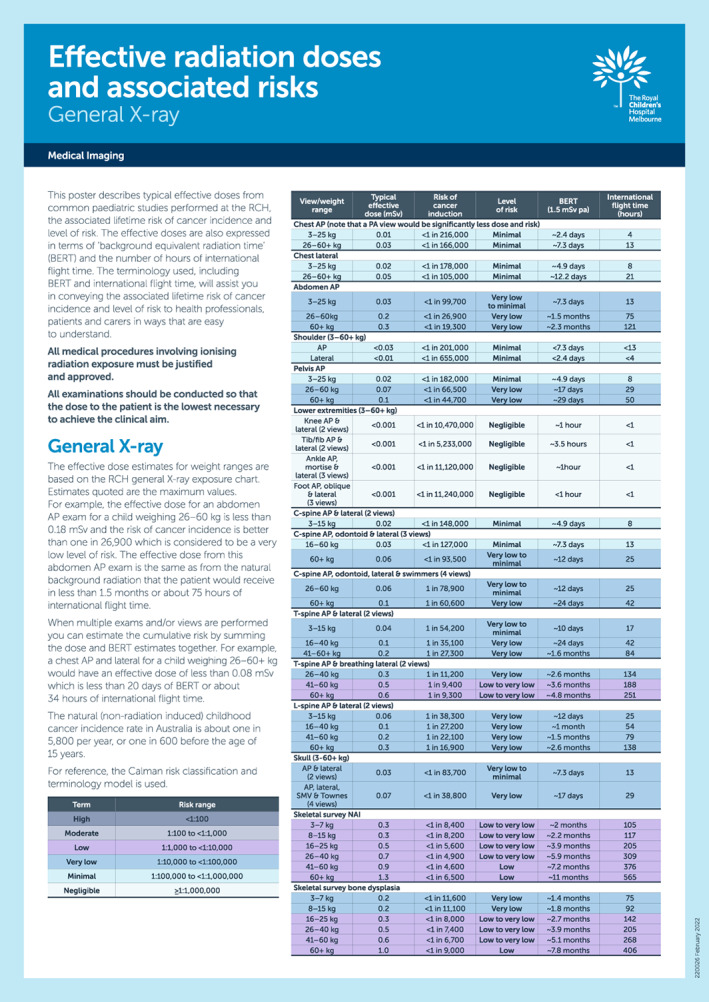
Typical effective doses, risk of cancer incidence, level of risk, comparison with natural background equivalent radiation time (BERT) of 1.5 mSv per annum and comparison with international flight time for commonly performed for general radiography examinations at the RCH.

Table 4 lists the typical effective doses, highest BEIR VII lifetime risk of cancer incidence (rounded as appropriate) and the associated Calman risk category for the included 20 examinations. It was observed that the effective doses did not vary significantly between weight ranges for some examinations and therefore the data could be summarised as shown in Figure 1, providing a simple standardised approach for the communication of risk in a paediatric setting which includes the typical effective dose, risk of cancer incidence, level of risk, BERT and equivalent international flight time.

**Table 4:** Typical effective doses and lifetime risk of cancer incidence for general radiography examinations at the RCH.

Exam	Weight Range (kg)	Estimated Effective Dose (mSv)	Highest Cancer Incidence Risk (1 in)	Risk Category
Chest AP^†^	3–7	0.007	283,000	Minimal
	8–15	0.008	275,000	Minimal
	16–25	0.014	216,000	Minimal
	26–40	0.022	171,000	Minimal
	41–60	0.024	208,000	Minimal
	60+	0.033	166,000	Minimal
Chest Lateral	3–7	0.007	317,000	Minimal
	8–15	0.012	193,000	Minimal
	16–25	0.017	178,000	Minimal
	26–40	0.027	133,000	Minimal
	41–60	0.038	105,000	Minimal
	60+	0.047	116,000	Minimal
Abdomen AP	3–7	0.010	201,000	Minimal
	8–15	0.013	167,000	Minimal
	16–25	0.030	99,700	Very low
	26–40	0.130	29,600	Very low
	41–60	0.178	26,900	Very low
	60+	0.286	19,300	Very low
Shoulder AP	3–7	0.003	748,000	Minimal
	8–15	0.004	570,000	Minimal
	16–25	0.006	494,000	Minimal
	26–40	0.015	261,000	Minimal
	41–60	0.021	238,000	Minimal
	60+	0.028	201,000	Minimal
Shoulder Lateral	3–7	0.001	2,326,000	Negligible
	8–15	0.001	2,780,000	Negligible
	16–25	0.002	1,742,000	Negligible
	26–40	0.003	1,292,000	Negligible
	41–60	0.007	655,000	Minimal
	60+	0.008	698,000	Minimal
Pelvis AP	3–7	0.011	182,000	Minimal
	8–15	0.012	187,000	Minimal
	16–25	0.015	192,000	Minimal
	26–40	0.058	66,500	Very low
	41–60	0.065	74,500	Very low
	60+	0.124	44,700	Very low
Knee AP and Lateral	3–7	<0.001	10,470,000	Negligible
	8–15	<0.001	11,120,000	Negligible
	16–25	<0.001	13,670,000	Negligible
	26–40	<0.001	18,090,000	Negligible
	41–60	<0.001	20,900,000	Negligible
	60+	<0.001	43,820,000	Negligible
Tibia/Fibula AP and Lateral	3–7	<0.001	5,233,000	Negligible
	8–15	<0.001	5,559,000	Negligible
	16–25	<0.001	6,836,000	Negligible
	26–40	<0.001	6,029,000	Negligible
	41–60	<0.001	10,450,000	Negligible
	60+	<0.001	21,910,000	Negligible
Ankle AP, Mortise and Lateral	3–7	<0.001	20,930,000	Negligible
	8–15	<0.001	11,120,000	Negligible
	16–25	<0.001	27,350,000	Negligible
	26–40	<0.001	36,180,000	Negligible
	41–60	<0.001	87,610,000	Negligible
	60+	<0.001	125,200,000	Negligible
Foot AP, Oblique and Lateral	3–7	<0.001	20,930,000	Negligible
	8–15	<0.001	11,240,000	Negligible
	16–25	<0.001	74,030,000	Negligible
	26–40	<0.001	90,440,000	Negligible
	41–60	<0.001	219,030,000	Negligible
	60+	<0.001	292,140,000	Negligible
Cervical Spine AP and Lateral	3–7	0.011	174,000	Minimal
	8–15	0.014	148,000	Minimal
Cervical Spine AP, Odontoid and Lateral	16–25	0.021	137,000	Minimal
	26–40	0.026	147,000	Minimal
	41–60	0.034	127,000	Minimal
	60+	0.059	93,500	Very low
Cervical Spine AP, Odontoid, Lateral and Swimmers	26–40	0.039	97,800	Very low
	41–60	0.056	78,900	Very low
	60+	0.091	60,600	Very low
Thoracic Spine AP and Lateral	3–7	0.035	59,800	Very low
	8–15	0.041	54,200	Very low
	16–25	0.071	38,000	Very low
	26–40	0.103	35,100	Very low
	41–60	0.144	28,800	Very low
	60+	0.202	27,300	Very low
Thoracic Spine AP and Breathing Lateral	26–40	0.317	11,200	Very low
	41–60	0.445	9400	Low
	60+	0.595	9300	Low
Lateral Spine AP and Lateral	3–7	0.050	41,900	Very low
	8–15	0.058	38,300	Very low
	16–25	0.094	27,600	Very low
	26–40	0.131	27,200	Very low
	41–60	0.183	22,100	Very low
	60+	0.326	16,900	Very low
Skull AP and Lateral	3–7	0.025	83,700	Very low
	8–15	0.025	89,000	Very low
	16–25	0.026	105,000	Minimal
	26–40	0.026	139,000	Minimal
	41–60	0.028	149,000	Minimal
	60+	0.031	178,000	Minimal
Skull AP, Lateral, Townes and SMV^‡^	3–7	0.054	38,800	Very low
	8–15	0.054	41,200	Very low
	16–25	0.056	49,700	Very low
	26–40	0.054	67,000	Very low
	41–60	0.059	72,100	Very low
	60+	0.066	83,600	Very low
Skeletal Survey – Non‐Accidental Injury	3–7	0.245	8400	Low
	8–15	0.272	8200	Low
	16–25	0.464	5600	Low
	26–40	0.724	4900	Low
	41–60	0.869	4600	Low
	60+	1.321	6500	Low
Skeletal Survey – Bone Dysplasia	3–7	0.184	11,600	Very low
	8–15	0.205	11,100	Very low
	16–25	0.324	8000	Low
	26–40	0.489	7400	Low
	41–60	0.617	6700	Low
	60+	0.960	9000	Low

^†^ Anterior–Posterior.

^‡^ Submentovertex.

## Discussion

The typical effective doses for 20 of the most commonly performed paediatric general radiography examinations were estimated using the weight‐based imaging protocols employed at our institution. The effective doses were used to estimate and categorise associated age‐based stochastic risks with commonly used risk terminology to facilitate the communication of risk to patients and carers. This mirrored the approach used by the authors in their earlier publication for estimating age‐dependent organ and effective doses and the associated lifetime risk of cancer incidence and level of risk for the six most commonly performed nuclear medicine and PET procedures at RCH for children aged 0–18 years using published organ and effective dose coefficients and commonly used risk terminology.^10^

As noted in ICRP Publication 128, effective dose can be a useful tool for comparing doses related to stochastic effects from other radiological procedures, similar or otherwise, performed at other institutions provided that the populations involved are of similar age and patient sex.^25^ Using age‐ and patient sex‐specific risk factors such as those provided in BEIR VII allow us to estimate the risk of developing a cancer from a particular procedure, additional to the lifetime baseline risk.

Whilst Table 2 provides exposure parameters for certain weight ranges, it was found that in some instances, the effective dose and associated stochastic risk values were sufficiently similar that some weight ranges could be combined to simplify the presentation of information. The grouping where possible also allowed for brevity in formatting of the final data for presentation and use (Fig. 1).

There are significant limitations involved in using the currently available data and information such as those provided in PCXMC, ICRP and BEIR VII documents for estimating an individual's dose and associated risk. Individuals who differ considerably from the body size and shape assumed in the calculations will have significantly different effective doses and risk estimates than those presented in Table 4 and Figure 1.^25^ There are many uncertainties inherent in the factors used to estimate effective dose, with nominal risk coefficients unable to be applied to specific individuals.^1^ Future work may include patient cohort studies investigating patient exposures, exposure indices and establishing local Diagnostic Reference Levels.

The accuracy of the PCXMC program has been well validated and its limitations well documented.^26^, ^27^, ^28^, ^29^ Incident air kerma for each exposure was based on the PCXMC estimate from the tube current‐time product (mAs) with a reported accuracy of about 30% (two standard deviations).^26^

Another limitation is modelling and estimating the dosimetry for upper extremities. As noted earlier, our dose estimates for humerus, forearm and hand were obtained by normalising published data for the exposure parameters (i.e. correcting for differences in kVp and mAs to account for a different beam quantity). It was not possible to account for other differences such as total beam filtration, source to skin and source to detector differences as this information was not provided in the published literature.

The estimated effective doses and associated risks can be used as a guide, with caution, for patients whose weight is significantly below or above the median weight for their age. The information is presented in large weight ranges therefore, for these patient cohorts, healthcare professionals can use the estimates to provide an approximate magnitude of dose and risk.

It should be noted that the information presented is for a single occurrence of the relevant examination. Some patients may require multiple radiographic examinations over time and the risk of stochastic effects is cumulative (additive) over a person's lifetime.^2^, ^30^ The risk from each individual examination is unchanged (e.g. a second chest radiograph carries the same risk as the first chest radiograph assuming they are performed within a certain period of time due to changes in stochastic risk with age) but combined they can be used to estimate the cumulated lifetime risk from all their examinations. For personalised risk estimates in these situations, a medical physicist should be consulted.

It is important that every imaging examination or procedure is appropriately justified by a radiation medical practitioner which includes comparing the clinical benefit of performing the examination with the radiation related risks.^1^ These should be explained to the patient and carer as part of the consent process. Risks are often personalised by patients and carers, especially in stressful situations, so it is important the appropriate information is provided by healthcare professionals in a manner that is easily understood alongside the fact that the risk of not performing the examination may lead to a far more significant risk (e.g. missed diagnosis).^5^, ^31^, ^32^ A person's previous health care experiences, social factors, education, belief systems and values as well as any emotions being experienced at the time (e.g. fear, anger, frustration) can affect how a person evaluates the risk.^31^ Whilst a ‘one in a million’ level of risk may be perceived as a low (or negligible if using Calman's classification) risk by the medical and scientific community, there will be patients and carers who personalise that risk as they being that ‘one’.^31^ It should also be noted that not all patients and carers are numerically literate and may be confused by a ‘1 in ….’ statement. For example, misinterpreting a 1 in 5000 chance as being a greater risk than a 1 in 500 chance. Therefore, the healthcare professionals should ensure that the risk is explained to a level that the patient and carer can understand. The Calman classifications may be appropriate for patients and carers that are not numerically literate and may be applied to explaining the risks and benefits of other medical procedures (e.g. surgery and anaesthetics).

As a result of this study, Figure 1 has been produced, providing a useful tool for radiographers and radiologists when communicating with the patient and their carers before, during and the after the examination. It, or one similar, can also be used by referring clinicians and radiologists when weighing up the radiation risks with the clinical benefits of a general radiography examination, or other modality such as CT, for their patients.

## Conclusion

Typical effective doses from common paediatric general radiography examinations and the associated lifetime risk of cancer incidence and level of risk have been established for our institution. This work can be used to standardise the approach for communicating radiation dose and associated risk information in a paediatric setting. The information presented in this paper, namely Figure 1, can be used to convey risks to health professionals, patients and carers in ways that are easy to understand and compare with other everyday risks. Also, it can be used by referring clinicians, radiographers and radiologists when weighing up the radiation risks with the clinical benefits of the procedure for their patients.

## Data Availability

All data generated or analysed during this study are included in this published article.
